# CORRECTION

**DOI:** 10.1111/cas.14936

**Published:** 2021-06-04

In an article^1^ titled “Understanding intratumor heterogeneity by combining genome analysis and mathematical modeling” by Atsushi Niida, Satoshi Nagayama, Satoru Miyano, and Koshi Mimori, the authors would like to add the following sentence at the end of the legends for Figures 3 and 5.

“This image was obtained by modifying a figure, which originally appeared in our previous work.^53^”

The authors apologize for the error.
